# Plasma Treatment of Polymer Powder as an Effective Tool to Functionalize Polymers: Case Study Application on an Amphiphilic Polyurethane

**DOI:** 10.3390/polym11122109

**Published:** 2019-12-16

**Authors:** Rossella Laurano, Monica Boffito, Alessandro Torchio, Claudio Cassino, Valeria Chiono, Gianluca Ciardelli

**Affiliations:** 1 Department of Mechanical and Aerospace Engineering, Politecnico di Torino, Corso Duca degli Abruzzi 24, 10129 Torino, Italy; rossella.laurano@polito.it (R.L.); alessandro.torchio@polito.it (A.T.); valeria.chiono@polito.it (V.C.); gianluca.ciardelli@polito.it (G.C.); 2 Department of Surgical Sciences, Università degli Studi di Torino, Corso Dogliotti 14, 10126 Torino, Italy; 3 Department of Science and Technological Innovation, Università del Piemonte Orientale, Viale Teresa Michel 11, 15121 Alessandria, Italy; claudio.cassino@uniupo.it

**Keywords:** poly(ether urethane), plasma treatment, stimuli-responsiveness, Toluidine Blue O assay, polymer functionalization, carbodiimide chemistry

## Abstract

Plasma treatment is a widely applied, easy, fast, and highly reproducible surface modification technique. In this work powder plasma treatment was exploited to expose carboxylic groups along the backbone of a water soluble polymer. Specifically, a custom-made amphiphilic poly(ether urethane) containing Poloxamer^®^ 407 blocks (*M*_w_ = 54,000 Da) was first synthesized and its powders were plasma treated in the presence of Acrylic Acid vapor. To maximize –COOH group exposure while preventing polymer degradation, different Ar gas flow rates (i.e., 10, 30, and 50 sccm) were investigated. Upon gas flow increase, significant polymer degradation was observed, with a 35% molecular weight reduction at 50 sccm Ar flow rate. On the other hand, the highest number of exposed carboxylic groups (5.3 × 10^18^ ± 5.5 × 10^17^ units/g_polymer_) was obtained by setting gas flow at 10 sccm. Hence, a gas flow of 10 sccm turned out to be the best set-up to maximize –COOH exposure while preventing degradation phenomena. Additionally, upon plasma treatment, no detrimental effects were observed in the thermoresponsiveness of polymer aqueous solutions, which was ensured by Poloxamer^®^ 407 blocks. Therefore, the newly developed technology here applied on an amphiphilic poly(ether urethane) could pave the way to the tailored design of a plethora of different multifunctional hydrogels.

## 1. Introduction

Plasma is a partially or totally ionized gas formed under specific conditions and composed of ions, electrons, and free radicals, which may potentially interact with contact materials [1]. Plasma treatment is an extremely powerful method for material surface modification, as it offers the possibility to modify material surface properties, without altering their bulk characteristics, and can be applied to a huge variety of polymers, metals, and ceramics. Furthermore, this technique is advantageous as it is highly versatile (different types of plasma gases can be used), environmentally friendly (avoiding the use of solvents), fast, and potentially scalable. In the biomedical field, plasma treatment is a widely employed strategy for the modification of biomaterial-based scaffolds, aiming at enhancing their wettability, biocompatibility, and cell adhesion properties. In this regard, the literature reports many works describing the key role exerted by surface functionalization in the improvement of biomaterial biological response. For instance, in 2011 Luna et al. investigated the influence of plasma treatment on fibroblast adhesion and proliferation on chitosan-based membranes [2]. Later, Khorasani et al. reported a higher nerve cell adhesion on poly(l-lactic acid) and poly(lactic-*co*-glycolic acid) films treated with an oxygen plasma [3], meanwhile Alves et al. showed an enhanced protein absorption on oxygen plasma treated polymeric films [4]. In another work, Hotchkiss et al. investigated the effect of plasma treated titanium on macrophage activation and cytokine production, proving that higher surface wettability resulted in an improved healing response [5]. Beyond this application, plasma treatment has been also exploited to expose reactive groups on material surface for the further grafting of specific bioactive moieties. For instance, Choi et al. exploited oxygen plasma treatment to generate peroxides on polyurethane films, which were later used for acrylic acid grafting [6]. In another work, Lee et al. plasma treated polymeric films in the presence of acrylic acid to expose carboxylic groups for the subsequent grafting of collagen [7]. Similarly, Sartori et al. grafted gelatin or poly(l-lysine) on the surface of poly(ester urethane) films exposing –COOH functional groups [8]. More recently, with the aim to design a bioengineered cardiac patch, poly(ester urethane)-based 3D printed scaffolds were surface functionalized with gelatin or laminin upon acrylic acid grafting/polymerization through plasma [9]. In a different approach, a cationized gelatin layer was deposited on poly(lactic acid)-based nanofibrous membranes previously treated with an oxygen plasma to introduce carboxylic groups as anionic moiety [10]. Polydimethylsiloxane (PDMS) membranes were also functionalized by a two-step Argon/Acrylic acid plasma treatment followed by conjugation with chitosan rose Bengal through carbodiimide chemistry, with the aim to confer antimicrobial ability to PDMS [11].

Another application of this technique, which has attracted the interest of both industry and research in the last decades, is the plasma treatment of powders. However, modification of particle surface is generally complex due to the need for uniform particle surface modification while avoiding aggregation phenomena. Hence, powder plasma treatment can be successfully applied if combined with a mixing apparatus for powders. Based on its efficiency, inexpensiveness, and environmental compatibility, plasma treatment of powders has found widespread application in the development of powder additives for printing inks, paints, thermoplastics, coating, and cosmetics, as recently thoroughly surveyed by Arpagaus et al. [12].

In the perspective of future biomedical applications, powder plasma treatment could allow the exposure of a very large number of functional groups on the particle surface, as a consequence of their large surface area per volume of particles. However, based on the available literature, plasma treatment has always been applied on materials already processed into their final configuration, such as porous scaffolds, dense films, membranes, particles, and devices. To the best of our knowledge, no work has ever reported the use of this technology to modify virgin materials, e.g., polymeric powders before the processing in their final shape. Such an approach could be of interest in the design of multi-functional materials, to be further processed into biomedical devices/scaffolds.

This work reports one possible application of powder plasma treatment for the preparation of multifunctional hydrogels, as an example of the potentiality of the technique. In this context, powder plasma treatment could be exploited to expose functional moieties on the surface of a water soluble polymer particulate with predefined granulometry, with no detrimental effects on material water solubility and capability to form hydrogels. The introduced functional groups along the polymer backbone could:

(i) provide the resulting hydrogels with a specific stimuli-sensitiveness (e.g., acid or basic pH sensitivity when amines or carboxylic groups are exposed, respectively);
(ii) allow chemical crosslinking upon addition of crosslinking agents (e.g., genipin when amines are exposed along polymer backbone);
(iii) allow the grafting of biomolecules, thus making the hydrogels bioactive (i.e., able to drive specific cell behaviors).

Additionally, the introduction of plasma treatment within the productive line of a hydrogel could facilitate and accelerate its scale-up production towards industrialization and marketability.

**Figure 1:**
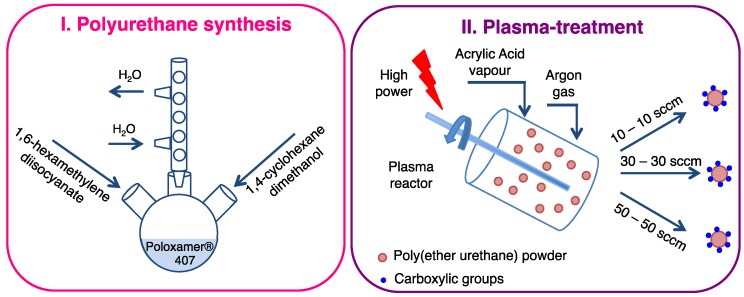
Schematic representation of the two main steps needed for the design of an amphiphilic poly(ether urethane) exposing a tunable amount of carboxylic groups: (I) polyurethane synthesis; (II) powder plasma treatment.

In more detail, in this work powder plasma treatment was performed on particulates based on a custom-made water soluble poly(ether urethane) (PEU) (Figure 1) with the aim to expose carboxylic groups. Specifically, an amphiphilic poly(ether urethane) was first synthesized starting from a commercial triblock copolymer (i.e., Poloxamer^®^ 407) as macrodiol, an aliphatic diisocyanate (1,6-hexamethylene diisocyanate), and a cyclic functional group-free chain extender (1,4-cyclohexanedimethanol). Then, PEU powders were plasma treated according to a two-step procedure, i.e., surface activation in the presence of Argon gas and continuous power supply followed by –COOH group exposure in the presence of Acrylic Acid vapor and alternated power supply.

However, only few studies have previously reported how plasma process parameters may affect functionalization degree and material degradation [13,14].

Based on that, in this work the influence of gas flow rate during plasma treatment on polymer chemistry was investigated. Specifically, PEU powders with a controlled average diameter were plasma treated by setting different Argon gas flow rates to maximize the exposure of carboxylic groups while preserving the polymer from degradation. The optimized treatment parameters were selected based on carboxylic group quantification and polymer molecular weight determination. To this purpose, attenuated total reflectance Fourier transform infrared spectroscopy, Toluidine Blue O colorimetric assay, proton nuclear magnetic resonance spectroscopy and size exclusion chromatography analyses were performed. To the best of our knowledge, this is the first time that the Toluidine Blue O colorimetric assay has been adapted to the quantification of carboxylic groups exposed on water soluble polymers. 

We have previously reported that [15,16,17] aqueous solutions of the here-studied amphiphilic PEU, containing Poloxamer^®^ 407 as building block, undergo temperature driven sol-to-gel transition by micelle self-assembly. In this work, the effect of plasma treatment on PEU thermosensitivity was studied, by estimating the critical micellar temperature of aqueous solutions of the modified polymer.

In conclusion, after powder plasma treatment, the PEU showed unaltered water solubility and thermoresponsiveness, while it exposed new carboxylic functionalities. Hence, powder plasma treatment could pave the way to the design of a new plethora of multifunctional hydrogels. As an example, the here-developed plasma treated PEU could find application in the preparation of hydrogels responsive to a double stimulus (temperature and pH). Alternatively, the carboxylic groups could be exploited for the grafting of specific moieties conferring bioactivity or crosslinking ability to the hydrogel. 

## 2. Materials and Methods

### 2.1. Materials

Before the synthesis, reagents (i.e., macrodiol, diisocyanate, and chain extender) were treated to remove residual water content and stabilizers according to the methods proposed by Pontremoli, Boffito et al. [18]. Briefly, Poloxamer^®^ 407 (P407, poly(ethylene oxide)-poly(propylene oxide)-poly(ethylene oxide) PEO-PPO-PEO triblock copolymer, *M*_n_ 12,600 Da, 70% *w*/*w* PEO) was dried at 100 °C for 8 h while keeping low pressure (i.e., 200 mbar) and then cooled down at room temperature (RT) under vacuum. 1,6-hexamethylene diisocyanate (HDI) was distilled under reduced pressure, meanwhile 1,4-cyclohexanedimethanol (CDM) was kept at RT under vacuum in a dessicator. Anhydrous 1,2-dichloroethane (DCE) was prepared by pouring the solvent over activated molecular sieves (3 Å, Sigma Aldrich, Milan, Italy) under constant nitrogen flow until use. P407, HDI, CDM, and dibutyltin dilaurate (DBTDL) as catalyst were purchased from Sigma Aldrich, Milan, Italy, while solvents were purchased from CarloErba reagents (Cornaredo, Italy) in their analytical grade.

### 2.2. Methods

#### 2.2.1. Synthesis Protocol

The P407-based poly(ether urethane) used in this work was synthesized under nitrogen atmosphere in a two step procedure according to the protocol recently published by Boffito, Pontremoli et al. [16]. Briefly, P407 was first dissolved in DCE at 20% *w*/*V* concentration and equilibrated at 80 °C; then, HDI was added to P407 solution at 2:1 molar ratio. The prepolymerization reaction continued for 150 min after the addition of a catalytic amount of DBTDL (0.1% *w/w* with respect to the macrodiol). Then, the mixture was cooled at 60 °C and the isocyanate-terminated prepolymer was chain extended by adding CDM (3% *w/V* in DCE) at 1:1 molar ratio with respect to P407. This second step proceeded for 90 min; then, the system was cooled down at RT and the reaction was terminated through the addition of anhydrous methanol. Subsequently, the synthesized PEU was collected by precipitation in petroleum ether (4:1 volume ratio) and dried overnight under the fume hood. Then, the polymer was dissolved at 20% *w/V* in DCE and purified in a mixture of diethyl ether/methanol (98:2 *V/V*) at 5:1 volume ratio with respect to DCE volume. Finally, the polymer was collected by centrifugation (Hettich, MIKRO 220R, Tuttlingen, Germany) at 0 °C and 6000 rpm for 20 min, dried overnight at RT under a fume hood, and stored at 4 °C under inert atmosphere. 

Hereafter, the synthesized PEU will be referred to with the acronym CHP407, where C, H, and P407 identify the chain extender, the diisocyanate, and the macrodiol, respectively.

#### 2.2.2. Plasma Treatment on CHP407 Powder

**Figure 2:**
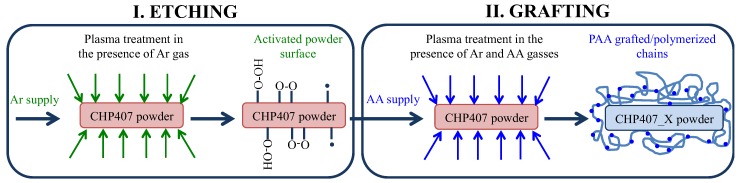
Schematic representation of the plasma treatment process: (I) etching phase in the presence of Ar gas to create free radicals on powder surface and (II) grafting step in the presence of Ar gas and Acrylic Acid vapor to expose carboxylic groups.

With the final aim of introducing carboxylic groups along polymer chains, plasma treatment was conducted on CHP407 powder using a plasma reactor (Diener electronic, Ebhausen, Germany) equipped with a rotary system as schematically reported in Figure 1. In detail, CHP407 powders were first sifted to collect samples (5 g) with controlled average diameter (size < 500 μm) and put in the reactor chamber. Then, PEU powders were subjected to a two-step functionalization procedure [19,20]: (i) surface activation (etching) through argon (Ar) plasma to create free radicals on powder surface and (ii) plasma treatment in the presence of acrylic acid (AA) vapor for the polymerization/grafting of poly(acrylic acid) on the polymeric surface and the consequent exposure of carboxylic groups (Figure 2). To maximize the amount of exposed functional groups, plasma process was optimized in terms of Ar gas supply by setting 10, 30, and 50 sccm as gas flow during both steps. Specifically, the chamber was first filled at the considered gas flow rate (i.e., 10, 30, or 50 sccm) for 10 min; then, the activation phase was performed at 50 W direct power supply for 5 min, while keeping the Ar gas flow constant. Subsequently, the reactor was purged with AA vapor at 10 μL/min for 15 min. In this phase plasma was induced for 10 min at 200 W alternated power supply (9 Hz, Pulse ON 10 ms, Pulse OFF 1000 ms, Duty Cycle 0.01, mean power applied 22 W) to prevent polymer degradation [21,22]. Ar supply was kept constant during the entire process at the same flow selected for the etching phase. To remove residual by-products (i.e., not-polymerized acrylic acid), plasma treated powders were then solubilized in chloroform and precipitated in petroleum ether (5:1 volume ratio with respect to chloroform). Finally, they were dried overnight under the fume hood and stored at 4 °C under nitrogen atmosphere before use.

Hereafter, plasma treated powder will be referred to with the acronym CHP407_X, where X stands for the adopted gas flow rate (i.e., 10, 30, and 50 sccm) during the etching and the grafting/polymerization steps.

#### 2.2.3. Attenuated Total Reflectance Fourier Transform Infrared Spectroscopy

To assess the success of the synthesis and to verify the presence of exposed carboxylic groups after plasma treatment, attenuated total reflectance Fourier transform infrared (ATR-FTIR) spectroscopy was performed on both CHP407 and CHP407_X samples. Specifically, polymers were analyzed using a Perkin Elmer Spectrum 100 equipped with an ATR accessory (UATR KRSS, Perkin Elmer, Waltham, MA, USA) with diamond crystal. Spectra resulted from 32 scans in the spectral range from 4000 to 600 cm^−1^ (resolution 4 cm^−1^). Spectra were registered at RT in triplicate, analyzed using the Perkin Elmer Spectrum software (Waltham, MA, USA), and reported as averaged spectra.

#### 2.2.4. Size Exclusion Chromatography

To prove the successful synthesis of a high molecular weight polymer, size exclusion chromatography (SEC) analyses were first performed on CHP407 samples using an Agilent Technologies 1200 Series (Agilent Technologies, Inc., Santa Clara, CA, USA). Subsequently, the same analyses were also conducted on CHP407_X samples to assess possible degradation phenomena induced by the plasma treatment. In detail, the instrument was equipped with a refractive index (RI) detector and two Waters Styragel columns (HR1 and HR4, Waters Corporation, Sesto San Giovanni, Italy) conditioned at 55 °C. N,N-dimethylformammide (DMF, CHROMASOLV Plus, inhibitor-free, for HPLC, 99.9%, CarloErba Reagents, Cornaredo, Italy) added with 0.1% *w/V* lithium bromide (LiBr, Sigma Aldrich, Milan, Italy) was selected as mobile phase at 0.5 mL/min. Number average molecular weight ($\bar{M_{n}}$), weight average molecular weight ($\bar{M_{w}}$), and polydispersity index (D) were estimated using the Agilent ChemStation software (Agilent Technologies, Inc., Santa Clara, CA, USA) starting from a calibration curve based on poly(ethylene glycol) standards with peak molecular weight (*M*_p_) in the range 1000–200,000 Da. Samples were prepared by dissolving 2 mg of polymer in the mobile phase (1 mL) and then by filtering the solution through a 0.45 μm syringe filter (poly(tetrafluoroethylene) membrane, Whatman, part of GE Healthcare Life Sciences, Little Chalfont, UK). Analyses were performed in triplicate and $\bar{M_{n}}$, $\bar{M_{w}}$, and D reported as mean ± standard deviation, while molecular weight distributions were reported as averaged profiles.

#### 2.2.5. Toluidine Blue O Colorimetric Assay

Toluidine Blue O (TBO) colorimetric assay was performed on CHP407_X samples to quantify the exposed carboxylic groups by adapting to water soluble polymers the protocol reported by Barish et al. for surface characterization [23]. Specifically, plasma treated samples (20 mg) were first dissolved in a 500 μM TBO (Sigma Aldrich, Milan, Italy) aqueous solution (40 mL) previously adjusted at pH 10. Subsequently, the electrostatic coupling reaction between the cationic dye and exposed –COOH groups was continued for 12 h at RT. After that, samples were put in dialysis (cut off 10–12 kDa, Sigma Aldrich, Milan, Italy) against double distilled water (ddH_2_O) for 4 days to wash out unreacted TBO molecules and finally freeze-dried (ALPHA 2-4 LSC, Martin Christ, Osterode am Harz, Germany). CHP407 samples were treated according to the same protocol as control condition. Then, 10 mg of freeze-dried samples were dissolved in 1 mL of acetic acid/ddH_2_O 50/50 *V/V* for 30 min to allow the desorbing reaction of bonded/adsorbed TBO molecules. Finally, the polymer was separated by centrifugation at 10,000 rpm, 15 °C for 10 min and the extract absorbance was measured at 632 nm using an UV/Vis spectrophotometer (Lambda 25, Perkin Elmer, Waltham, MA, USA). Carboxylic groups were quantified by referring to a calibration curve based on TBO molecules dissolved at different concentrations (range 1–20 μM) in acetic acid/ddH_2_O 50/50 *V/V*. Analyses were performed in triplicate and results reported as mean ± standard deviation.

#### 2.2.6. Proton Nuclear Magnetic Resonance Spectroscopy

To demonstrate the capability of the modified TBO colorimetric assay to effectively quantify the amount of exposed carboxylic groups on water soluble polymers, –COOH group quantification was also indirectly performed through Proton Nuclear Magnetic Resonance (^1^H-NMR) spectroscopy. To this aim, benzylamine (BA, Sigma Aldrich, Milan, Italy) was first grafted as signaling molecule to the –COOH groups exposed on CHP407_X (obtained by treating CHP407 powder at the optimized Ar flow rate) via carbodiimide chemistry. BA grafting was carried out in two steps: (i) carboxylic group activation at acidic pH and (ii) amide bond formation between PEU-COOH and BA-NH_2_ at a pH higher than BA pKa (i.e., 9.3). In detail, plasma treated samples were first dissolved at 0.5% *w/V* in an aqueous solution of 1-ethyl-3-(3-dimethylaminopropyl) carbodiimide and N-hydroxysuccinimide (5 and 1.25 mg/mL, respectively) for 20 h at 5 °C. Then, pH was adjusted to 5 and the activation reaction proceeded for 3 h at 5 °C. At the end of this step, BA (0.25 *mg/mL* in ddH_2_O) was added to the mixture at 10:1 molar ratio with respect to quantified carboxylic groups through TBO assay and the pH was adjusted to 12. The grafting reaction was carried on for 24 h at RT under magnetic stirring and stopped by adjusting the pH at 7.4. Finally, samples were put in dialysis (cut off 10–12 kDa, Sigma Aldrich, Milan, Italy) against ddH_2_O for 7 days to completely remove unbound BA and freeze-dried. Then, samples were analyzed using an Avance III Bruker NMR spectrometer (Bruker Italia S.r.l., Milan, Italy) equipped with an 11.75 T superconducting magnet (500 MHz ^1^H Larmor frequency). NMR spectra were recorded using Bruker BBFO direct probe (Bruker Italia S.r.l., Milan, Italy) and controlling samples’ temperature at 300 K using Bruker BVT 3000 unit (Bruker Italia S.r.l., Milan, Italy). BA quantification was carried out by measuring the area of the peak at 7.3 ppm in BA-grafted spectra and comparing it to that of a reference sample of free BA. In addition, to verify the repeatability of plasma treatment, ^1^H-NMR analyses were performed on plasma treated CHP407_X samples obtained from two independent procedures. For ^1^H-NMR analysis, 10 mg of BA-grafted plasma treated CHP407_X (CHP407_X_BA) were dissolved in 750 μL of deuterated dimethyl sulfoxide (d6-DMSO, >99.5%, Sigma Aldrich, Milan, Italy). Deuterated DMSO residual proton signal at 2.5 ppm was used as a reference for ^1^H chemical shift. ^1^H-NMR spectra were recorded averaging 12 scans with 10 s of relaxation delay. Control samples (CHP407_BA) were prepared and analyzed according to the same protocol. 

#### 2.2.7. Critical Micellar Temperature Estimation

In order to investigate whether plasma treatment could affect polymer thermoresponsiveness, the critical micellar temperature (CMT) of CHP407_X powder (obtained by treating CHP407 at the optimized Ar flow rate) was estimated according to the protocol proposed by Alexandridis et al. [24]. CHP407 samples were subjected to the same protocol as control condition. Specifically, samples were dissolved at 0.1% *w/V* concentration in physiological saline solution (0.9% *w/V* NaCl). Then, the fluorescent dye 1,6-diphenyl-1,3,5-hexatriene (DPH, Sigma Aldrich, Milan, Italy), previously dissolved at 0.4 mM in methanol, was added to each sample at 10 μL/mL concentration. Dye-containing samples were subjected to a controlled heating from 5 to 40 °C at 1 °C/step. Each step consisted of sample incubation at the set temperature for 5 min followed by UV/Vis spectroscopy (Lambda 25, PerkinElmer, Waltham, MA, USA) within the spectral range 330–400 nm (resolution 1 nm). Finally, CMT was estimated by considering the first inflection of the sigmoidal curve obtained by plotting the measured absorbance at 356 nm vs. temperature [15,17].

#### 2.2.8. Statistical Analysis

Statistical analysis was performed using GraphPad Prism version 8.0 for MacOsX (GraphPad Software, La Jolla, CA, USA; www.graphpad.com). Two-way ANOVA analysis followed by Bonferroni’s multiple comparison test was used to compare results. The statistical significance of each comparison was assessed according to Boffito et al. [15].

## 3. Results and Discussion

### 3.1. Poly(ether urethane) Chemical Characterization

**Figure 3:**
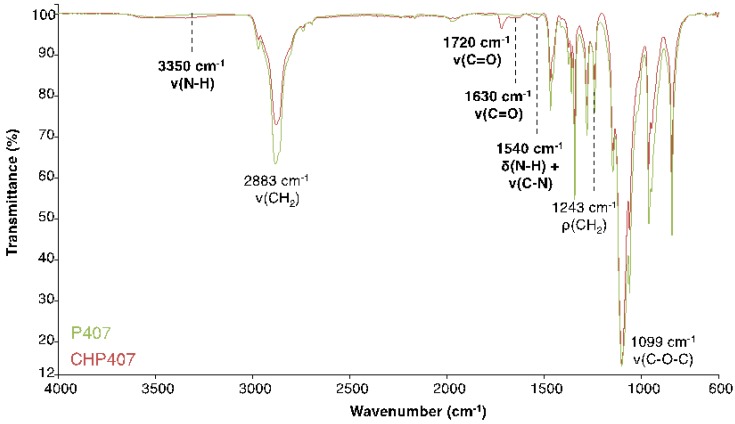
Attenuated total reflectance Fourier transform infrared (ATR-FTIR) spectra of Poloxamer^®^ 407 (green) and CHP407 (red). The bands that proved the success of the synthesis (i.e., urethane bond formation) are highlighted in bold.

The success of poly(ether urethane) synthesis was first confirmed through ATR-FTIR spectroscopy showing the presence of the typical absorption peaks of urethane bonds. These findings were in agreement with recently published data by Boffito et al. [25]. Figure 3 reports the ATR-FTIR spectra of Poloxamer^®^ 407 and the as-synthesized CHP407. 

The presence of Poloxamer^®^ 407 in the synthesized CHP407 was confirmed by the peaks at 2883, 1243, and 1099 cm^−1^, which can be assigned to the CH_2_ stretching and rocking vibrations and C–O–C stretching vibration, respectively [15].

On the other hand, the formation of urethane linkages was proved by the appearance of characteristic bands at: 1720 and 1630 cm^−1^, attributed to the stretching vibration of free and H-bonded carbonyl groups, respectively; 1540 cm^−1^, ascribed to the bending N–H vibration and the stretching of the C–N bonds and 3350 cm^−1^, assigned to the stretching vibration of N–H bonds. However, the peak at 1630 cm^−1^ may be also generated by the presence of urea bonds, which could be formed as byproduct of the first step, according to the work recently published by Laurano et al. [17] and Qin, Jiang et al. [26]. Nevertheless, the possible presence of a small amount of urea bonds is not expected to affect the successful synthesis of a high molecular weight poly(ether urethane).

The synthesis of a high molecular weight poly(ether urethane) was further proved by the increase in polymer number average molecular weight ($\bar{M_{n}}$) and weight average molecular weight ($\bar{M_{w}}$) compared to the initial Poloxamer^®^ 407 (Table 1). Additionally, PEU low polydispersity index (D) indicated a narrow molecular weight distribution and thus a good control over the polymerization process.

### 3.2. Chemical Characterization of Plasma Treated Poly(ether urethane)

**Figure 4:**
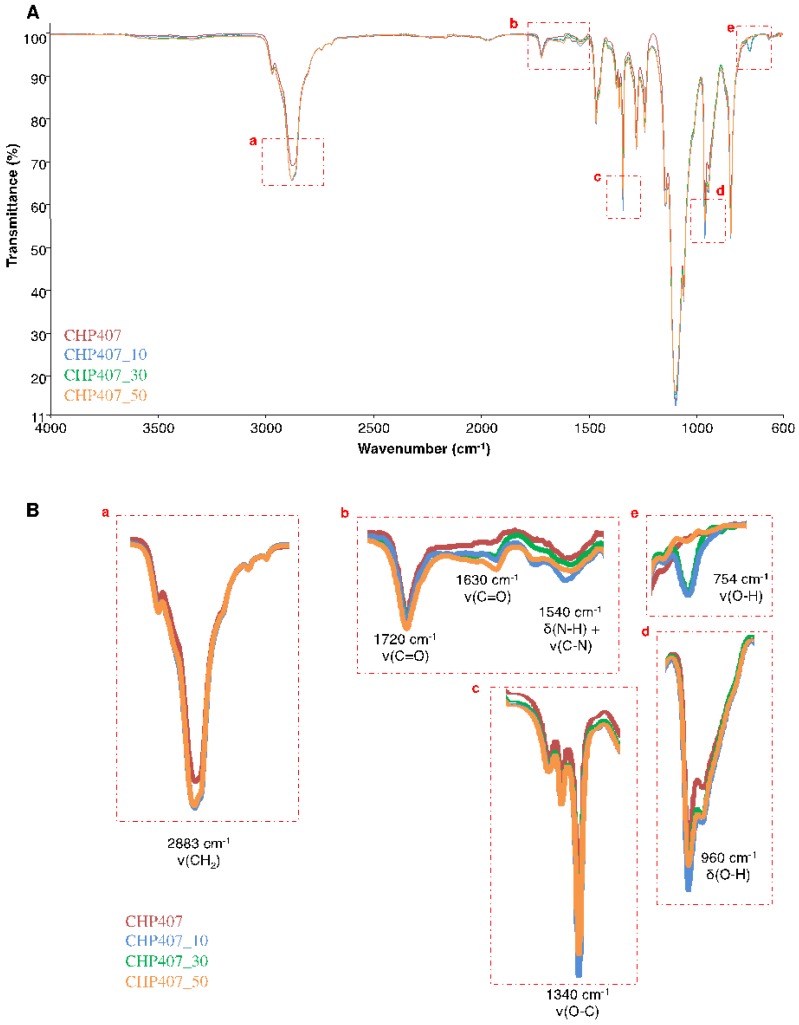
(**A**) ATR-FTIR spectra of untreated (CHP407) and plasma treated (CHP407_10, CHP407_30, and CHP407_50) samples; (**B**) bands proving the success of the plasma treatment are highlighted in the red boxes and reported as magnifications.

To verify the integrity of urethane bonds and the successful exposure of carboxylic groups after plasma treatment, ATR-FTIR analyses were performed on CHP407_X samples. Spectra were then compared to CHP407 as control condition (Figure 4A,B). 

During the etching phase of plasma treatment, an excessive interaction between the formed gas plasma and the polymeric chains could result in polymer degradation. This phenomenon is even more evident upon an increase in Ar gas flow rate due to the formation of a higher number of free radicals that can interact with polymer powder. 

As reported in Figure 4A, irrespective of tested Ar gas flow conditions, CHP407_X spectra showed the characteristic bands attributed to the urethane bonds (i.e., at 1720 and 1630 cm^−1^ C=O stretching vibrations, at 1540 cm^−1^ N–H bending and C–N stretching vibrations and at 3350 cm^−1^ N–H stretching vibration), thus suggesting that plasma treatment did not affect the integrity of urethane bonds. On the contrary, a slight decrease in the absorption intensity of the peak ascribed to the C–O–C stretching vibration at 1099 cm^−1^ was observed in CHP407_30 and CHP407_50 spectra (Figure 4B). This evidence suggested that slight degradation phenomena occurred when powders were treated at high gas flow rate. In fact, by increasing the gas volume subjected to the plasma irradiation, a higher number of ions and free radicals form, leading to a stronger interaction with polymer chains. However, being ATR-FTIR spectroscopy a qualitative analysis, no clear conclusion can be achieved on detrimental effects induced by plasma treatment on CHP407 powder. On the other hand, irrespective of plasma conditions, the success of the treatment was proved by the increased absorbance intensity of the bands attributed to the carboxylic groups and to the CH_2_ stretching vibration at 2883 cm^−1^ ascribed to polymerized acrylic acid (Figure 4B). Specifically, the comparison between CHP407 and CHP407_X ATR-FTIR spectra evidenced an increase in the absorbance intensity of the peaks at 1630 and 1340 cm^−1^ ascribed to the C=O and O–C stretching vibrations, respectively, and at 960 cm^−1^ attributed to the –OH bending vibration. In addition, CHP407_30 and CHP407_10 spectra showed the appearance of a new peak at 754 cm^−1^ ascribed to the O–H stretching vibration, further confirming the exposure of carboxylic groups. Finally, the complete removal of not-polymerized acrylic acid from all considered samples was proved by the absence of the bands at 629 and 634 cm^−1^ attributed to the =C–H stretching vibration.

As reported in literature, plasma treatment could potentially affect the integrity of treated polymer bonds depending on the adopted process conditions (e.g., continuous or alternated wave discharge, pressure, selected gas, gas flow rate) [28]. Moreover, this effect is even more evident when the treatment is performed on powders, instead of large surfaces, as in this condition the surface area subjected to the effect of the gas plasma is maximized. In this work, the correlation between the adopted gas flow rate during both the etching and the grafting phases and potential polymer degradation has been thoroughly investigated. To this purpose, changes in polymer molecular weight were studied through size exclusion chromatography analyses. Number average molecular weights ($\bar{M_{n}}$), weight average molecular weights ($\bar{M_{w}}$), and polydispersity indexes (D) are reported in Table 2.

Based on the reported mean values, only the treatment performed at 50 sccm Ar flow rate resulted in considerable plasma-induced degradation phenomena, with a statistically significant decrease of both $\bar{M_{n}}$ and $\bar{M_{w}}$ compared to the starting CHP407 (i.e., approx. a 35% and 26% decrease in $\bar{M_{n}}$ and $\bar{M_{w},}$ respectively). Moreover, a slight increase in the polydispersity index of CHP407_50 samples was also measured. 

**Figure 5:**
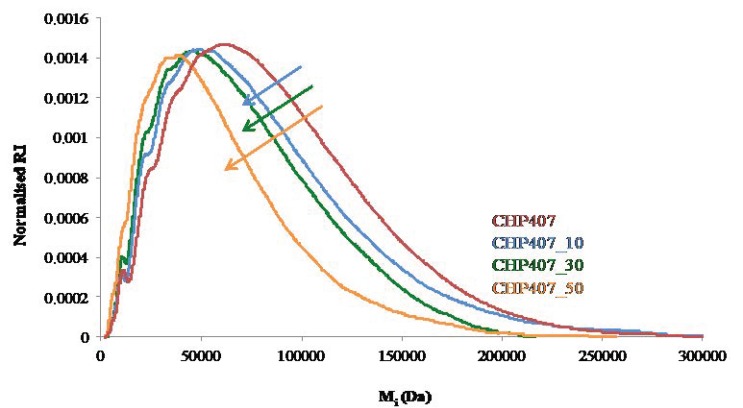
Molecular weight distribution profiles (normalized refractive index (RI) vs. molecular weight of each polymeric component composing the sample (*M*_i_)) of untreated (CHP407) and plasma treated poly(ether urethane) powder (CHP407_X, X = 10, 30, or 50 sccm). Each profile resulted from the average of three different analyses.

To thoroughly investigate changes in polymer molecular weight, the molecular weight distribution profiles of untreated and plasma treated samples are compared in Figure 5.

As shown in Figure 5, upon an increase in the set Ar gas flow during the entire plasma process, the maximum of the molecular weight distribution profiles of plasma treated samples slightly moved to lower molecular weights compared to the control (CHP407). However, no significant decrease in molecular weight was registered in CHP407_10 and CHP407_30 with respect to CHP407 (blue and green arrows, respectively). On the other hand, plasma treating CHP407 powders at 50 sccm Ar flow rate resulted in detrimental effects on polymer molecular weight (orange arrow, significant decrease with respect to CHP407, CHP407_10, and CHP407_30). Hence, the slight differences observed among CHP407, CHP407_10, and CHP407_30 fall within the typical margin of error which characterizes SEC analyses (up to 10% [27]), whereas in the case of CHP407_50 clear signs of plasma induced poly(ether urethane) degradation were detected. 

### 3.3. Carboxylic Group Quantification

The number of carboxylic groups, exposed along polymer backbone through plasma treatment in the presence of acrylic acid vapor, was first quantified by Toluidine Blue O (TBO) colorimetric assay. This colorimetric test has been widely validated in literature for the quantification of –COOH groups exposed on the surface of water insoluble dense and porous constructs [9,29,30]. However, to the best of our knowledge, this is the first time TBO assay has been adapted to quantify the functional groups exposed on water soluble polymers. 

**Figure 6:**
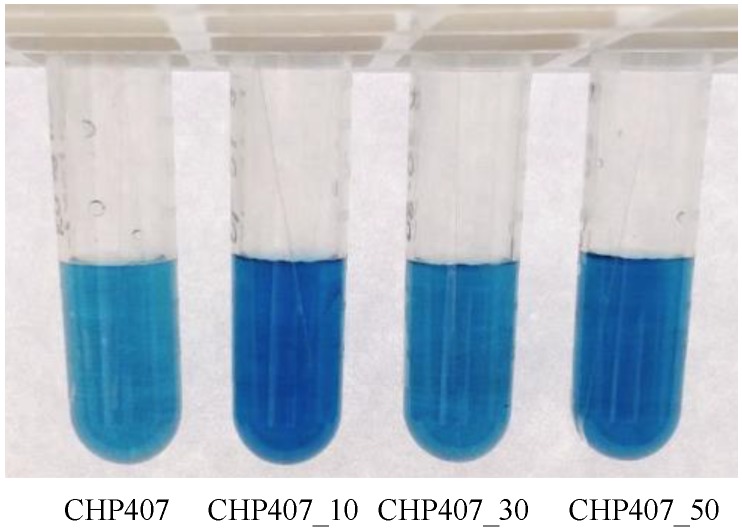
Toluidine Blue O (TBO) colorimetric assay performed on CHP407 powder (control condition) and on samples plasma treated with acrylic acid vapor at different Ar gas flow rates (i.e., 10, 30, and 50 sccm). The light blue color in CHP407 sample marks adsorbed TBO molecules, while dark blue in CHP407_X indicates the presence of TBO molecules grafted to –COOH groups, in addition to adsorbed ones.

As a consequence of the electrostatic interactions existing between TBO molecules and exposed carboxylic groups, CHP407_X samples exhibited a dark blue color after the desorbing reaction (Figure 6). However, also control samples (CHP407) treated with TBO assay showed a light blue color as a consequence of partial TBO adsorption by polymer chains. Nevertheless, the greater color intensity of CHP407_X samples with respect to CHP407 ones qualitatively confirmed the successful exposure of acidic groups in all tested conditions. In addition, the different color intensity obtained for CHP407_10, CHP407_30, and CHP407_50 suggested the introduction of a different number of carboxylic groups based on the set Ar gas flow rate. 

**Figure 7:**
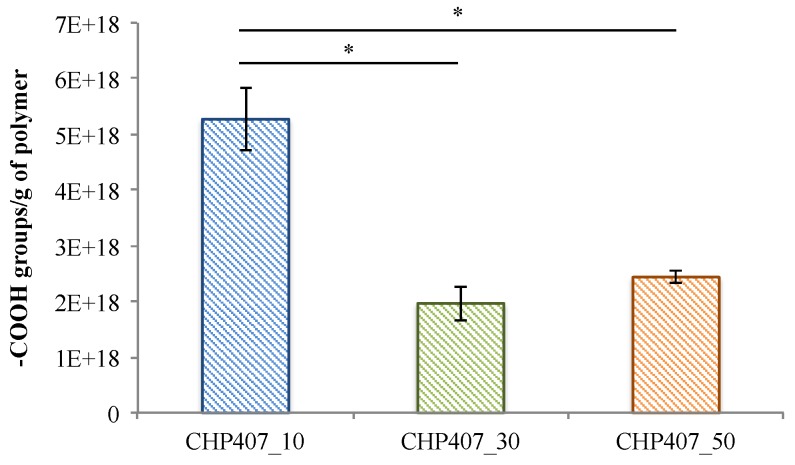
Quantification of exposed carboxylic groups on CHP407_X samples through Toluidine Blue O colorimetric assay. Analyses were performed in triplicate on samples belonging to three different batches. * *p* < 0.05.

Assuming that one TBO molecule electrostatically interacts with one carboxylic group, the total amount of –COOH units/g of polymer was calculated through the quantification of desorbed TBO molecules. To verify the intra- and inter-treatment repeatability of the –COOH-exposure procedure, TBO assay was performed in triplicate on three different batches of CHP407_10, CHP407_30, and CHP407_50. The amount of exposed carboxylic groups on CHP407_10, CHP407_30, and CHP407_50 is summarized in Figure 7. 

The highest number of exposed acidic groups was achieved by setting the Ar gas flow at 10 sccm, both in the etching and grafting phases (significantly higher number of –COOH/g_polymer_ in CHP407_10 with respect to CHP407_30 and CHP407_50). Indeed, the increase in gas flow rate hinders the achievement of a high degree of vacuum within the chamber (i.e., pressure approx. 0.5 mbar vs. 0.2 mbar during the plasma treatment of CHP407_50 and CHP407_10, respectively), which could invalidate the success of the treatment. Specifically, performing the plasma treatment at a higher pressure consequently leads to a decreased monomer residence time within the plasma reactor [31]. In addition, the presence of a higher volume of compressed gas also brings an increased temperature in the reactor chamber, which could lead to a partial powder melting and, as a consequence, to a decrease in the exposed superficial area. Therefore, an Ar gas flow rate of 10 sccm turned out to be the most effective condition leading to the highest amount of exposed carboxylic groups, while preserving the polymer powder against degradation/undesired melting. On the other hand, no differences were observed in the number of acidic groups exposed by setting the Ar gas flow rate at 30 or 50 sccm. 

**Figure 8:**
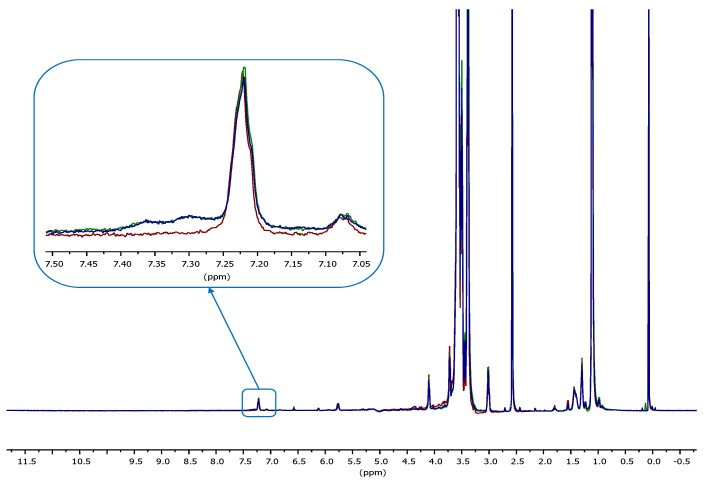
Proton nuclear magnetic resonance (^1^H-NMR) spectra of two benzylamine (BA)-grafted CHP407_10 batches (CHP407_10_BA_A and CHP407_10_BA_B, green and blue spectra, respectively) and a control sample (CHP407_BA, red spectrum). Magnified insert highlights differences between the spectra.

To further validate the results of TBO colorimetric assay, samples treated by setting the optimized gas flow rate (i.e., CHP407_10) were also analyzed through proton nuclear magnetic resonance (^1^H-NMR) spectroscopy after the grafting of a signaling molecule. Specifically, acidic groups were indirectly estimated through the quantification of the amount of benzylamine (BA) grafted to –COOH groups exposed on CHP407_10 powder via carbodiimide chemistry. Benzylamine was selected as signaling molecule since the signals of its aromatic ring protons (i.e., at 7.3 ppm) can be easily distinguished from the characteristic bands of the poly(ether urethane). To this purpose, analyses were performed on samples belonging to two different batches (CHP407_10_BA_A and CHP407_10_BA_B) to verify the repeatability of the plasma process. ^1^H-NMR analysis was also carried out on a CHP407 sample subjected to the same functionalization protocol as control condition (CHP407_BA). Figure 8 reports ^1^H-NMR spectra of both untreated and plasma treated samples analyzed after carbodiimide reaction to graft benzylamine molecules.

The absence of BA signals at 7.3 ppm in CHP407_BA spectrum proved that the dialysis step after polymer functionalization completely removed not grafted BA. Hence, the signals in CHP407_10_BA_A and CHP407_10_BA_B spectra at 7.3 and 7.35 ppm clearly confirmed the grafting of BA molecules to exposed carboxylic groups. Using BA ^1^H-NMR spectrum as reference (data not shown), the amount of grafted BA to 10 mg of CHP407_10 samples was measured to be in the 0.12–0.13 μmol range, which corresponds to approx. 7.5 × 10^18^ units/g of polymer. Therefore, the measured amount of BA was in agreement with the number of –COOH units quantified through TBO assay, with slight differences ascribable to the processing method. These results further supported the validity of those obtained through TBO colorimetric test and demonstrated the consistent adaptation of this assay to the quantification of functional groups exposed on water soluble polymers.

### 3.4. Polymer Thermoresponsiveness Evaluation

**Figure 9:**
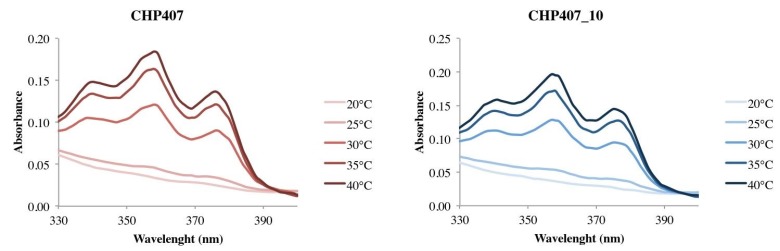
UV/Vis spectra of CHP407 (left) and CHP407_10 (right) solutions (0.1% *w/V*) added with 1,6-diphenyl-1,3,5-hexatriene (DPH) fluorescent dye recorded in the 330–400 nm spectral range upon heating up to 40 °C.

CHP407 and CHP407_10 thermoresponsiveness was evaluated through temperature-dependent UV/Vis spectroscopic analyses of their aqueous solutions added with a specific fluorescent dye (i.e., DPH). In fact, due to the presence of Poloxamer^®^ 407 as macrodiol, the here-synthesized PEU is expected to undergo chain organization into micelles as a consequence of temperature increase. Hence, polymeric structures with a hydrophobic core and a hydrophilic shell will form. For this reason, DPH can be used as an effective micellization marker as its absorbance intensity increases upon dispersion into a hydrophobic environment (e.g., micelle core). In this way, it is possible to estimate the critical micellar temperature (CMT), i.e., the temperature at which micelle nucleation begins. Specifically, in this study DPH assay was performed on both CHP407 and CHP407_10 aqueous solutions (0.1% *w/V*) to investigate the influence of carboxylic group exposure on chain organization capability in response to temperature variation. Figure 9 reports the absorbance profiles of both the analyzed systems within the range 330–400 nm recorded at different temperatures between 20 and 40 °C.

As illustrated in Figure 9, both systems did not show any absorbance peak up to 25 °C, suggesting that polymeric chains were still present in the form of unimers. Upon further temperature increase, the spectra of both CHP407 and CHP407_10 solutions showed increased absorbance intensities within the 330–400 nm spectral range, thus suggesting the formation of micelles in response to temperature changes. In detail, the estimated CMT for CHP407 and CHP407_10 aqueous solutions with 0.1% *w/V* concentration turned out to be 24.4 and 23.6 °C, respectively. The slightly lower CMT values measured for plasma treated sample can be correlated to the increased number of hydrogen bonds resulting from the exposure of –COOH groups along the polymer backbone. Indeed, hydrogen bonding has been reported to act as an interactive force triggering chain aggregation, in addition to hydrophobic interactions [31]. Nevertheless, the introduction of carboxylic groups through plasma treatment did not significantly alter the resultant polymer thermoresponsiveness. 

## 4. Conclusions

In this work plasma technology was used for the first time to successfully expose functional groups along the backbone of a hydrogel-forming material, without affecting its molecular weight, water solubility, and self-assembling potential. In detail, a custom-made amphiphilic poly(ether urethane) was first successfully synthesized and its powders with controlled average size (< 500 μm) were plasma treated in the presence of Ar gas and acrylic acid vapor with the final aim to introduce a considerable number of carboxylic groups. To maximize functionalization while avoiding polymer degradation, different Ar gas flow rates were tested (i.e., 10, 30, and 50 sccm). Results showed that degradation phenomena became increasingly evident with increasing gas flow rate from 10 to 50 sccm during the entire plasma process, with approx. 35% reduction in Number Average Molecular Weight for CHP407_50 samples respect to the virgin CHP407. Additionally, a lower number of carboxylic groups was achieved with increasing gas feed rate, in agreement with what reported by Iqbal et al. [22]. The amount of exposed carboxylic groups, measured by Toluidine Blue O colorimetric assay (here adapted to measure carboxylic groups exposed on water soluble materials) was 5.3 × 10^18^ ± 5.5 × 10^17^, 2 × 10^18^ ± 3 × 10^17^, and 2.4 × 10^18^ ± 1.1 × 10^17^ –COOH units/g for CHP407_10, CHP407_30, and CHP407_50, respectively. Hence, the highest number of –COOH groups was achieved by setting the Ar gas flow rate at 10 sccm. Correct quantification by Toluidine Blue O assay was confirmed by Proton Nuclear Magnetic Resonance spectroscopic analyses by measuring the amount of a signaling molecule (i.e., benzylamine) grafted to –COOH groups via carbodiimide chemistry. Thus, the modified TBO protocol proposed in this work represents a new and easy approach for the quantification of –COOH groups on water soluble materials. In addition, the exposure of –COOH groups in CHP407_10 did not alter polymer thermosensitivity, attributed to Poloxamer^®^ 407 building block, as demonstrated by the estimated Critical Micellar Temperature which turned out to be approx. the same as the control.

Hence, the plasma powder technology was successfully applied to introduce –COOH groups into a water soluble poly(ether urethane), while preserving its molecular weight and thermosensitivity. In the future, the here-developed amphiphilic poly(ether urethane) bearing carboxylic groups could find diverse applications in the biomedical field. For instance, thermo- and basic pH-sensitive hydrogels could be designed, undergoing changes in their network structure during deprotonation of –COOH groups in alkaline environment, modifying their permeability [32]. Biomimetic thermosensitive hydrogels could also be developed upon grafting of bioactive molecules. A proof of concept result was the successful grafting to the polymer of benzylamine via carbodiimide chemistry. Finally, chemically crosslinked hydrogels can be obtained by crosslinking reactions exploiting –COOH groups and using crosslinking agents, such as polycarbodiimides.

In conclusion, this work reported a novel approach based on powder plasma treatment for the design of functional polymers. The proof-of-concept application of powder plasma technology on a water soluble poly(ether urethane) evidenced that the following steps should be followed for the introduction of –COOH groups on polymers by this new method:

- Preparation of polymer powder with particle average dimension lower than 500 μm;
- Plasma treatment in the presence of Argon gas and Acrylic Acid vapors under selected gas flow conditions to avoid degradation phenomena, while allowing functionalization. Conditions should be optimized depending on polymer chemical nature;
- Verification of the results by Toluidine Blue O colorimetric assay and/or Proton Nuclear Magnetic Resonance spectroscopy.

The main novelty of this approach lies in the exploitation of the widely employed industrial plasma technology for the functionalization of polymers, which allows rapid and easy grafting of functional moieties, avoiding the use of potentially toxic organic solvents.

## Figures and Tables

**Table 1:** Number average molecular weight ($\bar{M_{n}}$), weight average molecular weight ($\bar{M_{w}}$), and polydispersity index (D) of P407 and CHP407 measured through size exclusion chromatography analyses (instrument error up to 10% [27], the table reports measurement uncertainty expressed as standard deviation of three analyses).

Sample	$\bar{M_{n}}\;(\mathrm{kDa})$	$\bar{M_{w}}\;(\mathrm{kDa})$	D
P407	8.6 ± 0.5	9.1 ± 0.4	1.2 ± 0.02
CHP407	34 ± 1.3	54 ± 1.4	1.6 ± 0.03

**Table 2:** Number average molecular weight ($\bar{M_{n}}$), weight average molecular weight ($\bar{M_{w}})$, and polydispersity index (D) of untreated (CHP407) and plasma treated poly(ether urethane) powder (CHP407_X, X = 10, 30, or 50 sccm). (instrument error up to 10% [27], the table reports measurement uncertainty expressed as standard deviation of three analyses).

Sample	$\bar{M_{n}}\;(\mathrm{kDa})$	$\bar{M_{w}}\;(\mathrm{kDa})$	D
CHP407	34 ± 1.3	54 ± 1.4	1.6 ± 0.03
CHP407_10	29 ± 0.8	49 ± 1	1.7 ± 0.02
CHP407_30	31 ± 0.5	54 ± 0.3	1.7 ± 0.03
CHP407_50	22 ± 1.8	40 ± 1.1	1.8 ± 0.05

## References

[1] Petlin D.G., Tverdokhlebov S.I., Anissimov Y.G. (2017). Plasma treatment as an efficient tool for controlled drug release from polymeric materials: A review. J. Control. Release.

[2] Luna S.M., Silva S.S., Gomes M.E., Mano J.F., Reis R.L. (2011). Cell adhesion and proliferation onto chitosan-based membranes treated by plasma surface modification. J. Biomater. Appl..

[3] Khorasani M.T., Mirzadeh H., Irani S. (2008). Plasma surface modification of poly (L-lactid acid) and poly (lactid-co-glycolic acid) films for improvement of nerve cells adhesion. Radiat. Phys. Chem..

[4] Alves C.M., Yang Y., Marton D., Carnes D.L., Ong J.L., Sylvia V.L., Dean D.D., Reis R.L., Agrawal C.M. (2008). Plasma surface modification of poly (D, L-lactid acid) as a tool to enhance protein adsorption and the attachment of different cell types. J. Biomed. Mater. Res. Part B.

[5] Hotchkiss K.M., Reddy G.B., Hyzy S.L., Schwartz Z., Boyan B.D., Olivares-Navarrete R. (2016). Titanium surface characteristics, including topography and wettability, alter macrophage activation. Acta Biomater..

[6] Choi H.S., Kim Y.S., Zhang Y., Tang S., Myung S.W., Shin B.C. (2004). Plasma-induced graft co-polymerization of acrylic acid onto the polyurethane surface. Surf. Coat. Technol..

[7] Lee S.D., Hsiue G.H., Chang P.C.T., Kao C.Y. (1996). Plasma-induced grafted polymerization of acrylic acid and subsequent grafting of collagen onto polymer film as biomaterials. Biomaterials.

[8] Sartori S., Rechichi A., Vozzi G., D’Acunto M., Heine E., Giusti P., Ciardelli G. (2008). Surface modification of a synthetic polyurethane by plasma glow discharge: Preparation and characterization of bioactive monolayers. React. Funct. Polym..

[9] Boffito M., Di Meglio F., Mozetic P., Giannitelli S.M., Carmagnola I., Castaldo C., Nurzynska D., Sacco A.M., Miraglia R., Montagnani S. (2018). Surface functionalization of polyurethane scaffolds mimicking the myocardial microenvironment to support cardiac primitive cells. PLoS ONE.

[10] Chen J.P., Su C.H. (2011). Surface modification of electrospun PLLA nanofibers by plasma treatment and cationized gelatin immobilization for cartilage tissue engineering. Acta Biomater..

[11] Ferreira A.M., Carmagnola I., Chiono V., Gentile P., Fracchia L., Ceresa C., Georgiev G., Ciardelli G. (2013). Surface mofication of poly (dimethylsiloxane) by two-step plasma treatment for further grafting with chitosan-Rose Bengal photosensitizer. Surf. Coat. Technol..

[12] Arpagaus C., Oberbossel G., von Rohr P.R. (2018). Plasma treatment of polymer powders—From laboratory research to industrial application. Plasma Process. Polym..

[13] Barton D., Bradley J.W., Steele D.A., Short R.D. (1999). Investigating radio frequency plasmas used for the modification of polymer surfaces. J. Phys. Chem. B.

[14] Atta A., Ali H.E. (2013). Structural and thermal properties of PTFE films by argon and oxygen plasma. Arab J. Nucl. Sci. Appl..

[15] Boffito M., Gioffredi E., Chiono V., Calzone S., Ranzato E., Martinotti S., Ciardelli G. (2016). Novel polyurethane-based thermosensitive hydrogels as drug release and tissue engineering platforms: Design and in vitro charcaterization. Polym. Int..

[16] Boffito M., Pontremoli C., Fiorilli S., Laurano R., Ciardelli G., Vitale-Brovarone C. (2019). Injectable thermosensitive formulation based on polyurethane hydrogel/mesoporous glasses for sustained co-delivery of functional ions and drugs. Pharmaceutics.

[17] Laurano R., Cassino C., Ciardelli G., Chiono V., Boffito M. (2020). Polyurethane-based thiomers: A new multifunctional copolymer platform for biomedical applications. React. Funct. Polym..

[18] Pontremoli C., Boffito M., Fiorilli S., Laurano R., Torchio A., Bari A., Tonda-Turo C., Ciardelli G., Vitale-Brovarone C. (2018). Hybrid injectable platforms for the in situ delivery of therapeutic ions from mesoporous glasses. Chem. Eng. J..

[19] Gancarz I., Pozniak G., Bryjak M., Frankiewicz A. (1999). Modification of polysulfone membranes. 2. Plasma grafting and plasma polymerization of acrylic acid. Acta Polym..

[20] Johnsen K., Kirkhorn S., Olafsen K., Redford K., Stori A. (1996). Modification of polyolefin surfaces by plasma-induced grafting. J. Appl. Polym. Sci..

[21] Susut C., Timmons R.B. (2005). Plasma enhanced chemical vapor depositions to encapsulate crystals in thin polymeric films: A new approach to controlling drug release rates. Int. J. Pharm..

[22] Iqbal M., Dinh D.K., Abbas Q., Imran M., Sattar H., Ahmad A.U. (2019). Controlled surface wettability by plasma polymer surface modification. Surfaces.

[23] Barish J.A., Goddard J.M. (2011). Topographical and chemical characterization of polymer surfaces modified by physical and chemical processes. J. Appl. Polym. Sci..

[24] Alexandridis P., Holzwarth J.F., Hatton T.A. (1994). Micellization of poly (ethylene oxide)-poly (propylene oxide)-poly (ethylene oxide) triblock copolymers in aqueous solutions: Thermodynamics of copolymer association. Macromolecules.

[25] Boffito M., Grivet Brancot A., Lima O., Bronco S., Sartori S., Ciardelli G. (2019). Injectable thermosensitive gels for the localized and controlled delivery of biomolecules in tissue engineering/regenerative medicine. Biomed. Sci. Eng..

[26] Qin J., Jiang J., Ye S., Wang S., Xiao M., Tao Y., Jie G., Meng Y. (2019). High performance poly (urethane-co-amide) from CO_2_-based dicarbamate: An alternative to long chain polyamide. RSC Adv..

[27] Trathnigg B., Meyers R.A. (2000). Size-exclusion chromatography of polymers. Encyclopedia of Analytical Chemistry.

[28] Liston E.M., Martinu L., Wertheimer M.R. (1993). Plasma surface modification of polymers for improved adhesion: A critical review. J. Adhes. Sci. Technol..

[29] Jeong J.O., Jeong S.I., Park J.S., Gwon H.J., Ahn S.J., Shin H., Lee J.Y., Lim Y.M. (2017). Development and characterization of heparin-immobilized polycaprolactone nanofibrous scaffolds for tissue engineering using gamma-irradiation. RSC Adv..

[30] Más B.A., Mara de Mello Cattani S., De Cássian Cipriano Rangel R., De Almeida Ribeiro G., Cruz N.C., De Lima Leite F., De Paula Nascente P.A., De Rezende Duek E.A. (2014). Surface characterization and osteoblast-like cells culture on collagen modified PLDLA scaffolds. Mater. Res..

[31] Aoki T., Kawashima M., Katono H., Sanui K., Ogata N., Okano T., Sakurai Y. (1994). Temperature-responsive interprenetrating polymer networks constructed with poly (acrylic acid) and poly (N, N-dimethylacrylamide). Macromolecules.

[32] Xu X.D., Zhang X.Z., Cheng S.X., Zhuo R.X., Kennerdy J.F. (2007). A strategy to introduce the pH sensitivity to temperature sensitive PNIPAAm hydrogels without weakening the thermosensitivity. Carbohydr. Polym..

